# Data on the regulation of moesin and merlin by the urokinase receptor (uPAR): Model explaining distal activation of integrins by uPAR

**DOI:** 10.1016/j.dib.2017.10.023

**Published:** 2017-10-16

**Authors:** Bernard Degryse, Mishan Britto, Chun Xu Shan, Robert G. Wallace, Keith D. Rochfort, Philip M. Cummins, Gerardene Meade, Ronan P. Murphy

**Affiliations:** Centre of Preventive Medicine, School of Health and Human Performance, Faculty of Science and Health, Dublin City University, Glasnevin, Dublin 9, Ireland

**Keywords:** ERM, ezrin/radixin/moesin, HAEC, Human aortic endothelial cells, uPAR, Urokinase receptor, Urokinase receptor, Moesin, Merlin, Angiogenesis, siRNA

## Abstract

The data presented herein are connected to our research article (doi: 10.1016/j.biocel.2017.04.012) [1], in which we investigated the functional connections between the urokinase receptor (uPAR), and the ezrin/radixin/moesin (ERM) proteins, moesin and merlin [1]. Firstly, a model of action is proposed that enlightens how uPAR regulates distal integrins. In addition, data show the effects of expressing wild-type moesin or permanently active T558D mutant of moesin on angiogenesis and morphology of human aortic endothelial cells (HAEC). Additional data compare the effects of urokinase (uPA, the main ligand of uPAR) on the same cells. Lastly, we provide technical data demonstrating the effects of specific siRNA for moesin and merlin on moesin and merlin expression, respectively.

**Specifications Table**

**Table:** 

Subject area	*Cell Biology*
More specific subject area	*Receptors and Signalling*
Type of data	*Images (microscopy, pictures of Western blot), Graphs, Figures*
How data was acquired	*Western blot, Densitometric analysis, Microscope (Olympus DP-50)*
Data format	*Analysed*
Experimental factors	*Transfection with specific siRNA either for moesin or merlin.*
	*Transfection with wild-type moesin or with T558D permanent active mutant of moesin.*
	*Treatment of human aortic endothelial cells with urokinase.*
Experimental features	*Actual knock down of moesin or merlin were verified after siRNA transfection.*
	*After treatment with or without urokinase, morphology of cells expressing wild-type moesin or mutant T558D was photographed.*
Data source location	*Dublin City University, Dublin, Ireland*
Data accessibility	*All data are included in this article*

**Value of the data**

• **Define the conditions for downregulating moesin or merlin expression with specific siRNA against moesin and merlin, respectively.**
• **Data are useful for understanding the effects of expressing mutants of moesin on cell morphology and angiogenesis.**
• **Show the effects of urokinase on angiogenesis and morphology of cells expressing mutants of moesin.**
• **Provide a model of action explaining how the urokinase receptor activates distal integrins.**

## 1 Data

**Fig. 1:**
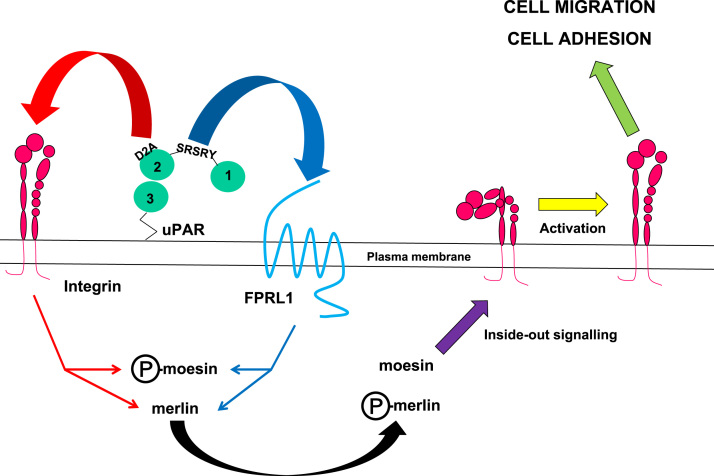
**Model explaining the distal activation of integrins by uPAR.** The binding of SRSRY sequence of uPAR to formyl peptide receptors such as FPRL1 (thick blue arrow) or the binding of D2A sequence located in domain 2 of uPAR to integrins (thick red arrow) initiates outside-in signalling converging towards phosphorylated moesin (P-moesin) and merlin (thin blue and thin red arrows), which results in the de-phosphorylation of moesin and phosphorylation of merlin (thick black arrow). This latter step initiates inside-out signalling (thick purple arrow) activating distal integrins (yellow arrow) that are involved in cell adhesion and migration (green arrow).

**Fig. 2:**
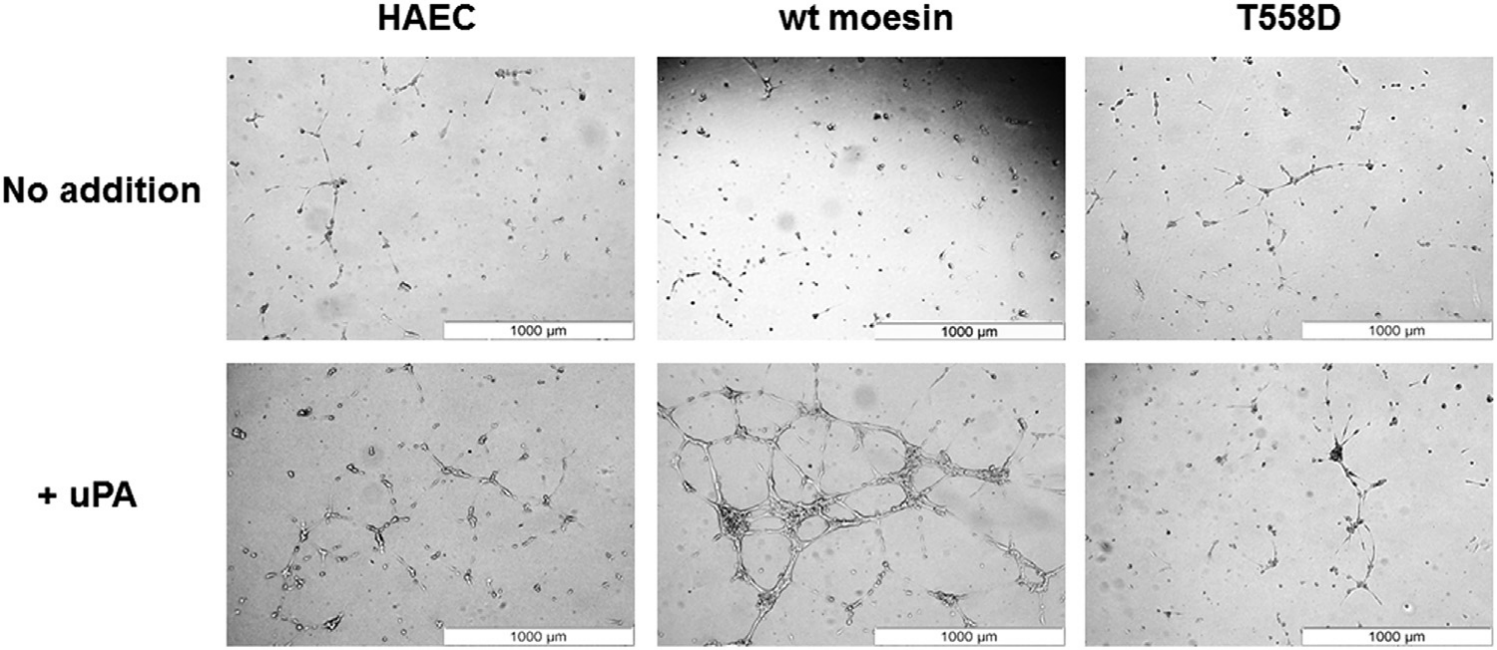
**Comparison of the effects of uPA on cell morphology of parental HAEC and HAEC transfected to express either wild-type moesin (wt moesin) or active T558D mutant**. Cells were seeded onto matrigel in the presence or in the absence of uPA (10 nM) for 12 h. Parental HAEC kept in the absence of uPA served as control. Then, low magnification pictures were taken (white scale bar 1000 μm). Quantification of these effects is shown in our research paper [1].

**Fig. 3:**
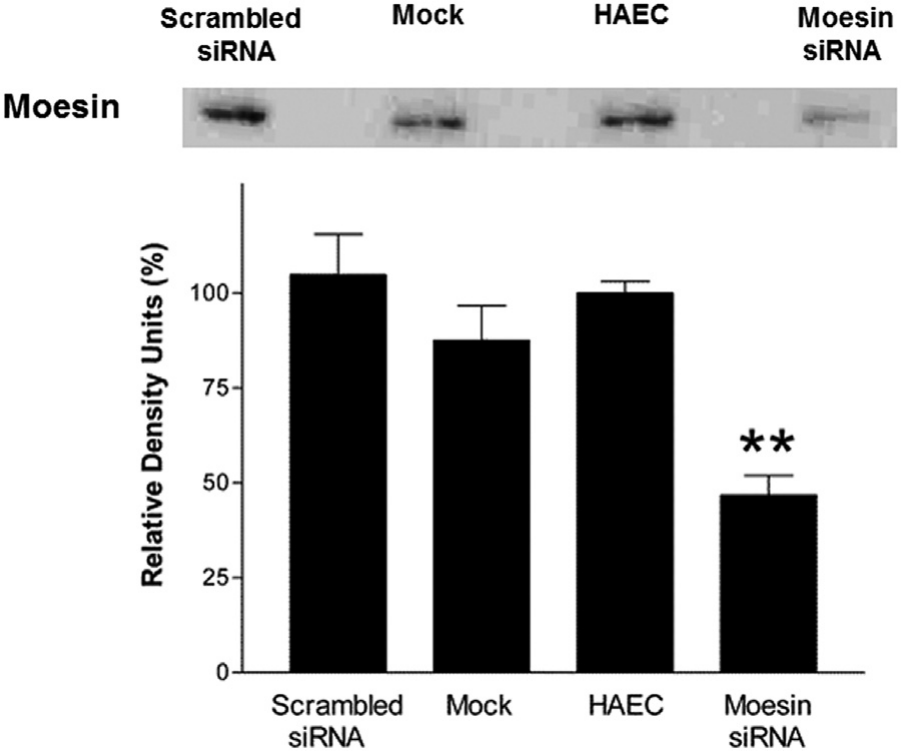
**Effects of moesin siRNA on expression of moesin.** Moesin expression was knocked down by treating HAEC with moesin siRNA and the level of expression of moesin was compared with that of untransfected HAEC. HAEC transfected without siRNA (mock) and HAEC transfected with unspecific scrambled siRNA (scrambled siRNA) served as positive controls. Then, cells were lysed and levels of expression of moesin were analysed by Western blotting. The upper picture shows moesin expression in each condition as indicated. This picture is representative of one out of three independent experiments. The lower bar graph represents the densitometric analysis (mean ± SD, n = 3) of the levels of expression of moesin. **P<0.01 compared to untransfected parental HAEC.

**Fig. 4:**
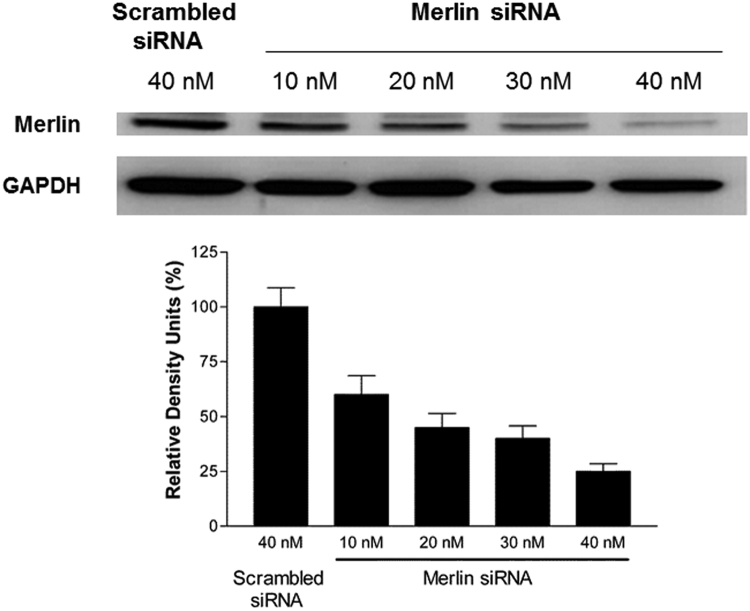
**Effects of merlin siRNA on expression of merlin in HAEC.** Merlin expression was knocked down by transfecting HAEC with increasing doses of merlin siRNA. HAEC transfected with unspecific scrambled siRNA (scrambled siRNA) served as control. Cells were lysed and levels of expression of merlin were analysed by Western blotting. The upper picture shows the levels of merlin expression after transfection with increasing doses of merlin siRNA as indicated. The lower picture displays the levels of glyceraldehyde 3-phosphate dehydrogenase (GAPDH) expression in the above conditions. These pictures are representative of one out of three independent experiments. The bar graph below represents the densitometric analysis (mean ± SD, n = 3) of the levels of expression of merlin normalised to GAPDH. **P<0.01 compared to scrambled siRNA.

uPAR is a membrane receptor involved in cell migration, adhesion and angiogenesis [2], [3], [4], [5], [6], [7], [8]. uPAR is a glycosyl-phosphatidyl-inositol receptor that is not connected to the intracellular compartment. Thereby, to induce intracellular signalling, uPAR interacts proximally with other membrane receptors such as the integrins [9], [10], [11]. Moesin and merlin belong to the family of ERM proteins. Moesin links membrane proteins to actin filaments permitting cell flexibility, and merlin regulates membrane receptors activity and signalling [12], [13], [14]. Fig. 1 represents a model built using experimental data from our research study [1], illustrating the functions of moesin and merlin. In line with this model, microscopy pictures show the effects of overexpressing wild-type moesin or permanently active mutant T558D on angiogenesis and morphology of human aortic endothelial cells (HAEC) (Fig. 2). The same HAEC transfected or not with wild-type moesin or mutant T558D, were also used to determine the effects of urokinase (uPA) on angiogenesis and cell morphology (Fig. 2). These qualitative data are completed by quantification of angiogenesis performed in our research paper [1]. Knocking down a protein using siRNA is a convenient method to investigate the function of that particular protein [15], [16], [17]. Here, we provide technical data for knocking down either moesin or merlin in HAEC using specific siRNA (Fig. 3, Fig. 4). Fig. 3 displays the effects of moesin siRNA compared to parental HAEC. In addition, HAEC transfected with scrambled siRNA or without siRNA (mock) served as negative controls. The effects of increasing doses of merlin siRNA were compared to the higher dose of scrambled siRNA used as negative control (Fig. 4).

## 2 Experimental design, materials and methods

### 2.1 Cell culture

HAEC were cultured according to the supplier (Promocell) in C-22020 endothelial cell growth medium MV plus SupplementMix® containing ECGS/H 0.4% (v/v), FBS 5% (v/v), EGF 10 ng/ml, hydrocortisone 1 μg/ml, 100 U/ml penicillin and 100 g/ml streptomycin. Only HAEC between passages 5–10 have been used for studies.

### 2.2 DNA transfection, Gene knockdown

According to manufacturer's instructions plasmid DNA was purified using the QIAGEN-tip HiSpeed kit. Nucleofector and a basic Nucleofector kit for endothelial cells (Lonza) were employed for transient transfection. 10^6^ HAEC and 3 µg DNA were added in 100 µl basic solution, transferred into an amaxa cuvette, and electroporated. Knockdown of moesin or merlin gene was performed using specific small interfering RNA (siRNA) for moesin (s8984, Ambion), and merlin/NF2 (s194647, Ambion). Scrambled siRNA (4390825, Applied Biosystems) served as negative control. Transfection was performed with 10^6^ HAEC and 4 µg of siRNA mixed in 250 µl of serum-free medium plus 7 μl of TransIT-siQUEST® reagent (Mirus), which were incubated for 20 min at room temperature, then seeded into 6-well plate containing 2 ml/well of fresh medium, and cultured for 2 days at 37 °C. Alternatively, transfection of 500,000 HAEC with 1–100 nM siRNA was realized with Labtech microporation unit (1000 V, 30 ms pulse width and 3 pulse number).

### 2.3 Microscopy

8,000 HAEC were seeded onto thick layer of matrigel (Corning) in cell culture media plus 0.5% of FCS, and cultured for 12 hours in the presence or in the absence of 10 nM uPA. Then, low magnification photographs (4 lens) were taken under the microscope (Olympus DP-50). Photographs shown in Fig. 2 are representative of one out of six independent experiments performed in triplicate.

### 2.4 Western blotting, densitometric analysis

HAEC were lysed in RIPA buffer: 20 mM Tris pH7.4, 150 mM NaCl, 1 mM Na_2_EDTA, 1 mM EGTA, 1% Triton X-100 (v/v), 2.5 mM sodium pyrophosphate, 1 mM β-glycerophosphate, 1 mM sodium orthovanadate, 1 µg/ml leupeptin, plus protease inhibitor mixture (Roche Diagnostics). Then, 50 µg of proteins were fractionated by SDS-PAGE using 10% acrylamide gel, and analysed by Western blotting. The separated proteins were transferred to nitrocellulose membranes, which were blocked by incubation for either 1 hour at room temperature or overnight at 4 °C in TBST pH 8.0, 10 mM Tris, 150 mM NaCl, 5% BSA (w/v), washed, and further incubated for 1 h with polyclonal antibody against moesin (sc-12895, Santa Cruz Biotechnology) or against merlin (ab2478, Abcam). Alternatively, blots were stripped using stripping buffer (Pierce), and incubated with anti-GAPDH antibody (ab8245, Abcam). Membranes were subsequently incubated for 1 hour with horseradish peroxidase-conjugated anti-rabbit antibody in 1% BSA-TBST, processed with Supersignal® West Pico (Pierce), and G-Box chemi system (Syngene). The densitometric analysis of the blots was performed using ImageJ (NIH) software.

### 2.5 Statistical analysis

Student's *t test* for pairwise comparisons of treatments was performed with the GraphPad Prism software.
